# AIF Overexpression Aggravates Oxidative Stress in Neonatal Male Mice After Hypoxia–Ischemia Injury

**DOI:** 10.1007/s12035-022-02987-0

**Published:** 2022-08-17

**Authors:** Tao Li, Yanyan Sun, Shan Zhang, Yiran Xu, Kenan Li, Cuicui Xie, Yong Wang, Yafeng Wang, Jing Cao, Xiaoyang Wang, Josef M. Penninger, Guido Kroemer, Klas Blomgren, Changlian Zhu

**Affiliations:** 1 Henan Children’s Neurodevelopment Engineering Research Center, Children’s Hospital Affiliated to Zhengzhou University, Zhengzhou, 450018 China; 2 Henan Key Laboratory of Child Brain Injury, Institute of Neuroscience and Third Affiliated Hospital, Zhengzhou University, Zhengzhou, 450052 China; 3 Center for Brain Repair and Rehabilitation, Institute of Neuroscience and Physiology, University of Gothenburg, 40530 Gothenburg, Sweden; 4 Department of Human Anatomy, School of Basic Medicine and Institute of Neuroscience, Zhengzhou University, Zhengzhou, 450052 China; 5 Centre of Perinatal Medicine and Health, Institute of Clinical Science, University of Gothenburg, 40530 Gothenburg, Sweden; 6 Institute of Molecular Biotechnology, Austrian Academy of Sciences, 1030 Vienna, Austria; 7 Department of Medical Genetics, Life Sciences Institute, University of British Columbia, Vancouver, Canada; 8 Centre de Recherche Des Cordeliers, Equipe Labellisée Par La Ligue Contre Le Cancer, Inserm U1138, Université de Paris Cité, Sorbonne Université, Institut Universitaire de France, Paris, France; 9 Metabolomics and Cell Biology Platforms, Institut Gustave Roussy, Villejuif, France; 10 Institut du Cancer Paris CARPEM, Department of Biology, Hôpital Européen Georges Pompidou, AP-HP Paris, France; 11 Pediatric Oncology, Karolinska University Hospital, Stockholm, Sweden; 12 Department of Women’s and Children’s Health, Karolinska Institutet, Stockholm, Sweden

**Keywords:** Sex difference, Apoptosis-inducing factor, Apoptosis, Oxidative stress, Hypoxia ischemia, Neonate

## Abstract

There are sex differences in the severity, mechanisms, and outcomes of neonatal hypoxia–ischemia (HI) brain injury, and apoptosis-inducing factor (AIF) may play a critical role in this discrepancy. Based on previous findings that AIF overexpression aggravates neonatal HI brain injury, we further investigated potential sex differences in the severity and molecular mechanisms underlying the injury using mice that overexpress AIF from homozygous transgenes. We found that the male sex significantly aggravated AIF-driven brain damage, as indicated by the injury volume in the gray matter (2.25 times greater in males) and by the lost volume of subcortical white matter (1.71 greater in males) after HI. As compared to females, male mice exhibited more severe brain injury, correlating with reduced antioxidant capacities, more pronounced protein carbonylation and nitration, and increased neuronal cell death. Under physiological conditions (without HI), the doublecortin-positive area in the dentate gyrus of females was 1.15 times larger than in males, indicating that AIF upregulation effectively promoted neurogenesis in females in the long term. We also found that AIF stimulated carbohydrate metabolism in young males. Altogether, these findings corroborate earlier studies and further demonstrate that AIF is involved in oxidative stress, which contributes to the sex-specific differences observed in neonatal HI brain injury.

**Supplementary Information:**

The online version contains supplementary material available at 10.1007/s12035-022-02987-0.

## Background

Hypoxic–ischemic encephalopathy (HIE) is a severe central nervous system injury caused by oxygen deprivation and limited blood flow in the neonatal brain. It is a major cause of mortality in neonates and can result in profound and devastating lifelong mental and physical disabilities, including cerebral palsy, seizures, and cognitive impairments in both term and preterm neonates [1, 2]. HIE is a global problem with an estimated incidence ranging from 1 to 8 per 1000 live births in developed countries to 26 per 1000 live births in underdeveloped countries [3]. Therapeutic hypothermia within 6 h of hypoxia–ischemia (HI) onset has been clinically shown to be a promising therapeutic intervention [4], but it only reduces the risk of death and disability by about 11%, meaning that up to 40% of the treated infants still develop neurological deficits [5]. Although erythropoietin treatment has demonstrated remarkable neuroprotection in infants [6, 7], the window of opportunity and optimal dosage is still controversial. Therefore, more in-depth research into neuronal cell death and the mechanisms of brain injury after HI is warranted in order to develop more effective therapies for preventing and treating neonatal brain injury.

The immature brain is particularly susceptible to HI injury [3, 8, 9]. Multiple mechanisms are involved in this process, including energy depletion, oxidative stress, excitotoxicity, and inflammatory responses, all of which lead to the activation of several distinct cell death pathways, including apoptosis, necrosis, necroptosis, ferroptosis, and autophagy [10–13]. During the normal development of the mammalian nervous system, apoptosis occurs extensively and has been observed in populations of developing neural precursor cells, differentiated neurons, and glial cells [14], or depression of apoptotic cell death may lead to neuroanatomic abnormalities and possibly to developmental disabilities [15, 16]. Thus, the immature brain is likely to be more susceptible to activation of apoptotic cell death pathways than the adult brain. Our previous studies have shown that apoptotic cell death accounts for a large portion of neuronal cell loss in neonatal HI injury [17] and that AIF, a principal component of the caspase-independent apoptotic cell death pathway, is a significant contributor to neuron loss induced by neonatal cerebral HI [18].

Under normal physiological conditions, AIF is a flavoprotein oxidoreductase located at the inner mitochondrial membrane [19] where it functions as a reactive oxygen species (ROS) scavenger targeting H_2_O_2_ [20] and in redox cycling with nicotinamide adenine dinucleotide phosphate [21]. Oxidative stress results from an imbalance between pro-oxidants and antioxidants in living cells [22], and many lines of evidence have suggested that oxidative stress is one of the major contributors to perinatal HI injury in newborns [23–25]. In Harlequin (Hq) mice, which have an 80% reduction in AIF expression [20], enhanced oxidative stress is seen in dying neurons. Therefore, AIF has been proposed to act as a free radical scavenger to inhibit apoptosis, and AIF deficiency might cause mitochondrial dysfunction resulting in disrupted oxidative phosphorylation and impaired neurogenesis of specific cell types. However, from the opposite perspective, whether upregulation of AIF can reduce oxidative stress and protect against neonatal HI injury is unknown.

Clinical evidence suggests that the mortality rate of male infants is higher than females, which suggests that sex is one of the risk factors in HIE [26]. Male infants exhibit increased risk for HI and display greater behavioral and cognitive disruption following HI injury compared to matched female counterparts [27]. Moreover, the therapeutic effect of hypothermia may be affected by sex because outcomes of HI in males tend to be worse than in females [27]. Animal studies utilizing models of neonatal HI support this difference and suggest that this sex discrepancy might be explained by sex-specific hormones, the presence of X-linked inhibitor of apoptosis protein in females, and sex-associated disparities in inflammatory responses [28, 29]. Besides the sex differences in mechanisms and outcomes of neonatal HI injury, the severity of injury varies by region and vulnerability [3]. Therefore, to further clarify the molecular mechanism of AIF in HIE, it is important to evaluate the influence of these differences on neonatal HI brain injury when AIF is upregulated.

Sex differences in neonatal HI brain injury were the focus of this study, and our results showed more severe brain injury in male AIF overexpressing mice (AIF hTg mice) compared with females, and males had more pronounced neuronal cell death and apoptotic cell death after HI. These results were related to lower antioxidant capacity and higher oxidative stress in males compared to females. AIF also significantly promoted neurogenesis in females in the long term and increased bioenergetic metabolism in males under physiological conditions.

## Materials and Methods

### Animals

**Fig. 1:**
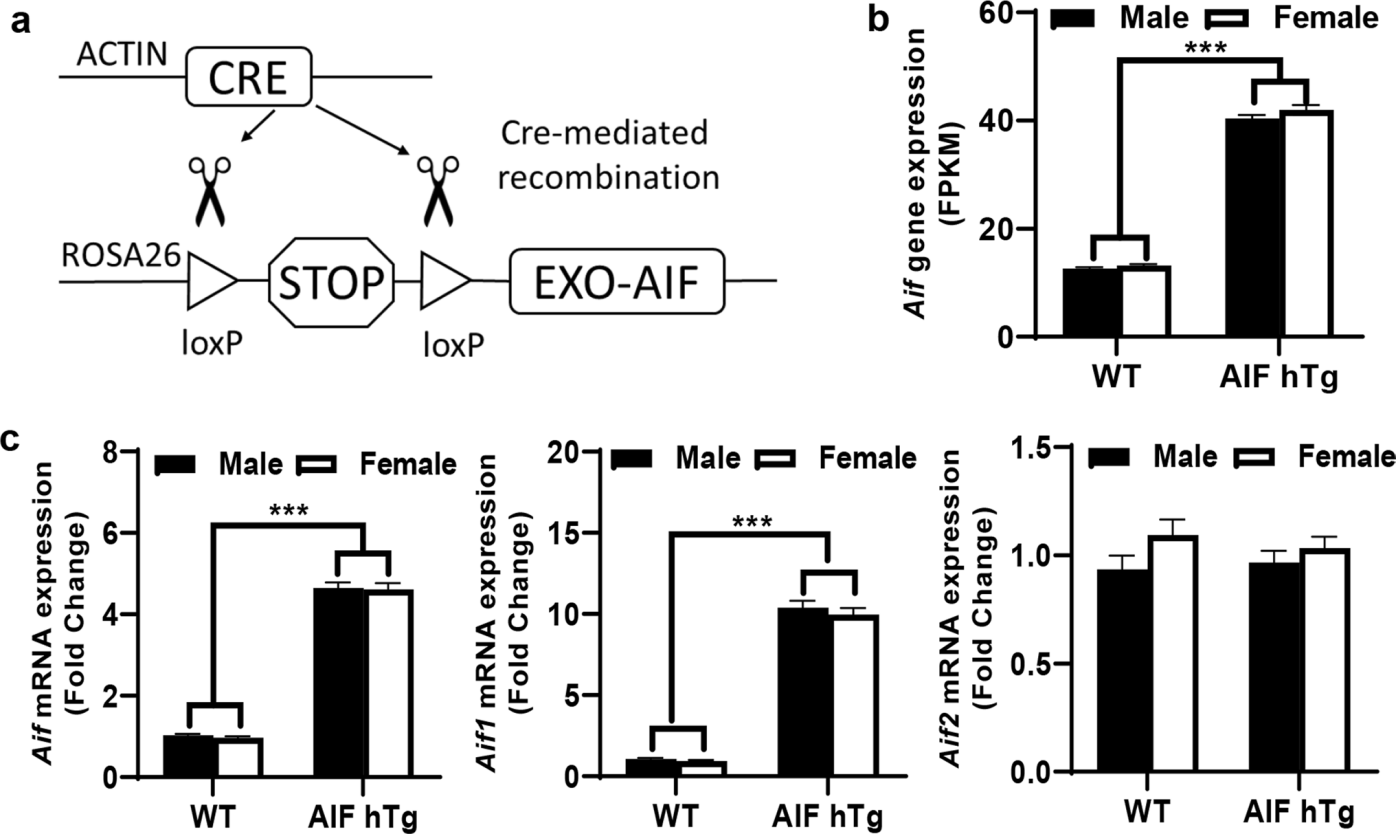
Determination of AIF overexpression in AIF hTg mice. **a** Schematic figure of AIF-Tg^flox/flox^-actin-Cre mice. **b** Based on the FPKM data from the RNA-seq analysis, *Aif* expression was significantly increased in AIF hTg mice compared to WT mice under physiological conditions (*n* = 6/group). **c** The mRNA expression of different variants was determined at 24 h post-HI using RT-qPCR (*n* = 6/group). The total transcription of the *Aif* gene in AIF hTg mice was significantly higher than in WT mice, and this upregulation only existed for *Aif1* and not for *Aif2*. Data are presented as the mean ± SEM and were analyzed using two-way ANOVA followed by Sidak’s post hoc test. ***p < 0.001

The Cre-lox recombination system was used to create the AIF overexpression mice, and the breeding pattern was consistent with our previous study [30]. The floxed mice with the insertion of the exogenous *Aif* gene at the Rosa26 gene locus were crossed with beta-actin-Cre mice (Fig. 1a). Both sexes of AIF-Tg^flox/flox^-actin-Cre (homozygous AIF hTg) and AIF Tg^+/+^-actin-Cre (wild-type, WT) mice with reasonable body weight (4.0–5.5 g) at postnatal day (P) 9 and with 5–8 pups per litter were used in this study. A total of 9 pups, including 6 males (3 WT) and 3 females (2 WT), were excluded because of death during hypoxia, and a total of 111 mouse pups were used for analysis. No statistical methods were used to predetermine sample size, and instead we based our experimental design on numbers reported in previous studies. All animals were allocated to the different experimental groups according to the different genotypes and were housed in a controlled temperature and pathogen-free environment under a 12-h light/dark cycle. All experimental procedures conformed to guidelines established by the Swedish Board of Agriculture (SJVFS 2019: 10) and were approved by the Gothenburg Animal Ethics Committee (112/2014). All animal experiments were performed in the Laboratory for Experimental Biomedicine of Gothenburg University and followed the guidelines of ARRIVE (Animal Research: Reporting in vivo Experiments). Genomic DNA was isolated from the tail sample, and PCR was performed for the genotyping. The primers for the AIF transgenic flox gene were 5′-GAG TTC TCT GCT GCC TCC TG-3′ (forward), 5′-AAG ACC GCG AAG AGT TTG TC-3′ (reverse for flox band, 215 bp), and 5′-CGA GGC GGA TAC AAG CAA TA-3′ (reverse for WT band, 322 bp), and the primer pair for the beta-actin-Cre gene was 5′-CTG CCA CGA CCA AGT GAC AGC AAT G-3′ (forward) and 5′-GCC TTC TCT ACA CCT GCG GTG CTA A-3′ (reverse) to produce an amplicon of 326 bp.

### HI surgery

Unilateral HI was induced in mice of both sexes on P9, which is roughly equivalent to the brain development of a term newborn infant, according to the Rice–Vannucci model [17]. Mice of both sexes were anesthetized with isoflurane (5% induction, 1.5–2.0% maintenance), and the duration of anesthesia and surgery was < 5 min. The right common carotid artery was permanently ligated. The wounds were infiltrated with xylocaine after the surgical procedure. Following a 1-h recovery in their dam’s cage, the pups were placed in a chamber perfused with a humidified gas mixture (10% ± 0.01% oxygen in nitrogen) for 40 min at 36 °C. After the hypoxic exposure, the pups were returned to their dams until sacrifice. Control pups were subjected to all procedures except HI.

### BrdU Administration

Bromodeoxyuridine (BrdU) (Roche, Mannheim, Germany, 5 mg/mL dissolved in 0.9% saline) was prepared fresh before use and injected intraperitoneally (50 mg/kg) on P8 and P9. The pups were continuously maintained without further disruption until P30 to collect brain tissues.

### Tissue Collection and Histology

Brain tissues were harvested at 24 h or 72 h after HI. For the AIF hTg mice under physiological conditions, brain tissues were harvested at P30. The sample harvesting and histological procedures were as described in our previous study [30]. These slides were used for immunofluorescence staining for BrdU and doublecortin (DCX).

### Fluoro-Jade Staining

After deparaffinization and rehydration, the sections were incubated with freshly prepared 0.06% potassium permanganate for 15 min and then rinsed in distilled water for 2 min. The sections were then incubated with 0.0004% Fluoro-Jade B (Merck Millipore, AG310-30MG) for 30 min in the dark at 21 °C and then washed with distilled water and mounted with ProLong Gold anti-fade reagent with DAPI (Invitrogen, P36931).

### Immunohistochemistry

Brain sections were deparaffinized in xylene and rehydrated in graded ethanol, and antigen retrieval was performed by heating the sections in 10 mM boiling sodium citrate buffer (pH 6.0) for 10 min and allowing them to cool for 30 min. Non-specific binding was blocked by incubating with 4% donkey or goat serum in PBS for 30 min, and endogenous peroxidase activity was blocked with 3% H_2_O_2_. The primary antibodies were as follows and were diluted in PBS and incubated with the sections overnight at 4 °C: monoclonal mouse anti-MAP2 (1:1,000 dilution, clone HM-2, Sigma, M4403), monoclonal mouse anti-MBP (1:500 dilution, clone SMI94, BioLegend, 836,504), polyclonal rabbit anti-cleaved caspase-3 (1:200 dilution, Asp175, Cell Signaling, 9661), and monoclonal rabbit anti-AIF (1:500 dilution, E20, Abcam, ab32516) antibodies. After the primary antibody incubation, the appropriate biotinylated secondary antibodies (1:200 dilutions, all from Vector Laboratories, Burlingame, CA, USA) were added to each section for 60 min at room temperature. The sections were visualized with a Vectastain Elite ABC HRP Kit (Vector Laboratories, PK-6100) and 0.5 mg/mL 3,3′-diaminobenzidine enhanced with ammonium nickel sulfate, β-D glucose, ammonium chloride, and β-glucose oxidase. After dehydrating with graded ethanol and xylene, the sections were mounted on coverslips with Vector mounting medium.

### Immunofluorescence and Microscopy

The deparaffinization, rehydration, and antigen recovery were performed as above. After blocking with 4% donkey serum in PBS for 30 min, the monoclonal mouse anti-3-NT (1.5:1000 dilution, 7A12AF6, Abcam, ab110282), polyclonal rabbit anti-DCX (1:500 dilution, Abcam, ab18723), or monoclonal mouse anti-BrdU (1:200 dilution, IIB5, Abcam, ab8955) antibodies were incubated with the sections overnight at 4 °C. After the primary antibody incubation, the donkey anti-mouse Alexa Fluor® 488 (1:500 dilution, Life Technology, A21202) or donkey anti-rabbit Alexa Fluor® 488 (1:500 dilution, Life Technology, A21206) secondary antibodies were added to each section for 120 min at room temperature. After washing, the sections were mounted on coverslips with ProLong Gold anti-fade reagent with DAPI (Invitrogen, P36931). For BrdU-DCX double staining, the mixed mouse anti-BrdU and rabbit anti-DCX primary antibodies, as well as mixed donkey anti-rabbit Alexa Fluor® 488 and donkey anti-mouse Alexa Fluor® 594 secondary antibodies, were used following the same staining procedure above.

Fluorescent staining was visualized using a Zeiss Axio Scan.Z1 digital slide scanner (Carl Zeiss, Germany), and panoramic images were processed using Zen software. For measuring the number of 3-NT-positive cells and the relative fluorescence units (RFUs) of 3-NT-positive areas in different brain regions, the whole brain sections, including both hemispheres, were scanned to obtain panoramic images using a 20 × objective lens. Three different sections with 50 interval sections were scanned for each brain sample. Three different regions of each section, including the cortex, cornus ammonis 1 (CA1), and striatum, were further captured within a defined area using Zen software. For measuring the number of BrdU-positive cells, the number of BrdU-DCX-positive cells, the DCX-positive area, and the RFUs of this area in the hippocampus, the whole hippocampal area of three sections was scanned at 250-μm intervals using the same scanner settings.

### Brain Injury Evaluation

Brain injury was evaluated based on microtubule-associated protein 2 (MAP2) and myelin basic protein (MBP) immunostaining. Both hemispheres of each section were measured using Micro Image (Olympus, Japan) after the staining. The MAP2-positive and negative tissue volume, the MBP-positive tissue volume of the cerebral subcortical white matter (SWM) area, and the neuropathological scores of the gray matter in different brain regions were assessed as described previously [30]_._ Briefly, the cortical injury was graded from 0 to 4 with 0 indicating the absence of observable injury and 4 confluent infarction. The injury in the hippocampus, striatum, and thalamus was assessed both with respect to hypotrophy (scored from 0 to 3) and injury/infarction (scored from 0 to 3), resulting in a total possible score of 22. All evaluations were carried out by an experienced investigator blinded to group assignment.

### Cell Counting, Positive Staining Area, and RFU Measurement

Area contours with fixed locations were drawn and measured in every 50th section. The section thickness was 5 μm. The active caspase-3-positive cells, AIF-positive nuclei, and Fluoro-Jade-positive cells were counted within a defined area (one visual field) of the cortex (100 × magnification), striatum (200 × magnification), CA1 (200 × magnification) (Fig. S1), and habenular nuclei (200 × magnification). The 3-NT-positive cells were counted in a fixed and same-sized area of the cortex (100 ×), CA1 (200 ×), and striatum (200 ×), and RFUs of the 3-NT-positive area were measured using the ImageJ software. BrdU-positive cells in the granular layer of the dentate gyrus were counted at 200 × magnification. The DCX-positive area and RFUs in the granular layer of the dentate gyrus (200 × magnification) were measured using ImageJ software. For the measurements using ImageJ, images were split into channels to transfer into gray mode. The positively stained area was determined by manually setting a threshold to include stained tissue, the integrated density of this positive area was measured, and the result was expressed as RFUs. All of the countings and measurements were carried out by investigators blinded to group assignment.

### Sample Preparation for Immunoblotting and ELISA Assay

The pups were sacrificed by decapitation at 24 h after HI. Tissue from the parietal cortex (including the hippocampus) in both hemispheres was rapidly dissected out and homogenized immediately on ice using a 2-ml Dounce tissue grinder set (Sigma, D8938), and an isolation buffer was added (15 mM Tris–HCl, pH 7.6, 320 mM sucrose, 1 mM dithiothreitol, 1 mM MgCl_2_, 3 mM EDTA-K, and 0.5% protease inhibitor cocktail (Sigma, P8340)). The procedures for cellular fraction isolation, including nuclear, cytosolic, and mitochondrial fractions, were as described in our previous study [30]. All fractions were kept at – 80 °C.

### Immunoblotting

Protein concentration was determined using the bicinchoninic acid method. A total of 65 µl of each sample was mixed with 25 µl of NuPAGE LDS 4 × sample buffer (ThermoFisher Scientific, NP0007) and 10 µl of NuPAGE Sample Reducing Agent (ThermoFisher Scientific, NP0004) and heated at 70 °C for 10 min. Samples were run on 4–12% NuPAGE Bis–Tris gels (Invitrogen) and transferred to reinforced nitrocellulose membranes (Bio-Rad). After blocking with 5% fat-free milk in TBST buffer (20 mM Tris, 150 mM NaCl, and 0.1% Tween 20, pH 7.6) for 60 min at room temperature, the membranes were incubated overnight with the following primary antibodies: rabbit anti-phospho-DRP1 (1:1000 dilution, Ser637, Cell Signaling, 4867), mouse anti-OPA1 (1:1000 dilution, BD Bioscience, 612,606), rabbit anti-FIS1 (1:500 dilution, FL-152, Santa Cruz, sc-98900), and mouse anti-VDAC1 (1:500 dilution, B-6, Santa Cruz, sc-390996). After washing, the membranes were incubated with peroxidase-labeled goat anti-rabbit IgG antibody (1:2000 dilution, Vector, PI-1000) or peroxidase-labeled horse anti-mouse IgG antibody (1:4000 dilution, Vector, PI-2000). Immunoreactive species were visualized using the SuperSignal West Pico PLUS Chemiluminescent Substrate (ThermoFisher Scientific, 34,580) and an LAS 3000 cooled CCD camera (Fujifilm, Japan).

### Total Antioxidant Capacity, Lipid Peroxidation, and Protein Carbonyl Content Assay

The homogenate of cortical brain tissue was used to measure total antioxidant capacity, lipid peroxidation, and protein carbonylation. The concentrations of total antioxidant capacity (Sigma-Aldrich, MAK187), malondialdehyde (MDA, Sigma-Aldrich, MAK085), and protein carbonyl content (Abcam, ab126287) were measured according to the manufacturer’s instructions. The concentration of total antioxidants was calculated as nmol per μL, MDA was calculated as nmol per mg protein, and the protein carbonyl content was calculated as nmol per mg protein. All measurements were performed by a person who was blinded to the grouping.

### RNA-seq Analysis

Cortical samples from both sexes of P9 WT and AIF hTg mice were prepared for RNA sequencing. Total RNA from each sample was extracted using an RNeasy Mini kit (Qiagen, 74,104), and the library preparation was done using an MGI Easy mRNA Library Prep Kit (BGI, Wuhan, China) following the manufacturer’s instructions. The sequencing library was used for cluster generation and sequencing on a BGISEQ-500 system (BGI) [31]. To explore the potential molecular mechanisms underlying the effects of AIF overexpression, Gene Set Enrichment Analysis (GSEA) was performed using the clusterProfiler package to identify enriched terms predicted to have a correlation with the Kyoto Encyclopedia of Genes and Genomes (KEGG) pathways. The criterion of *p* < 0.05 was considered statistically significant.

### RT-qPCR

Total RNA concentration and purity were determined using a Nanodrop spectrophotometer (Nanodrop Technologies, Wilmington, USA). One microgram of total RNA was reverse transcribed using the QuantiTect Reverse Transcription kit (Qiagen, 205,311). According to the manufacturer’s instructions, RT-qPCR was performed using the LightCycler 480 instrument (Roche Diagnostics, Mannheim, Germany) and the SYBR green (ThermoFisher Scientific, 0253) technique. The primers used in the qPCR reactions with the mitochondrial fission and fusion genes were designed by Beacon Designer software (PREMIER Biosoft) and were as follows: *Aif* (sense: 5′- TAT TTC CAG CCA CCT TCT TTC-3′, anti-sense: 5′-TTC ACC ATG TTG CCT CTT AC-3′), *Aif1* (sense: 5′-AGT CCT TAT TGT GGG CTT ATC-3′, anti-sense: 5′-GCA ATG GCT CTT CTC TGT T-3′), *Aif2* (sense: 5′-TTC TTA ATT GTA GGA GCA ACA GT-3′, anti-sense: 5′-CCC ATC ACT CTT TCA TTG TAT CT-3′), *Drp1* (sense: 5′-TGC TCA GTA TCA GTC TCT TC-3′, anti-sense: 5′-GGT TCC TTC AAT CGT GTT AC-3′), *Fis1* (sense: 5′-ATG AAG AAA GAT GGA CTG GTA G-3′, anti-sense: 5′-GGA TTT GGA CTT GGA GAC A-3′), *Opa1* (sense: 5′-CCT GTG AAG TCT GCC AAT-3′, anti-sense: 5′-TTA GAG AAG AGA ACT GCT GAA AT-3′), and *Sdha* (the reference gene) (sense: 5′-TTG CCT TGC CAG GAC TTA-3′, anti-sense: 5′-CAC CTT GAC TGT TGA TGA GAA T-3′). The relative expression levels of mRNAs were calculated according to the formula of 2^−(ΔΔCT)^.

### Statistical Analysis

GraphPad Prism 8.01 Software (GraphPad Software, San Diego, CA, USA) was used for all analyses. Comparisons between groups were performed by Student’s *t*-test, and data with unequal variance were compared with the Mann–Whitney *U*-test. Two-way ANOVA followed by Sidak’s post hoc test was used for multiple comparisons of data from more than two groups. Results are presented as means ± standard error of the mean (SEM), and *p* < 0.05 was considered statistically significant.

## Results

### AIF hTg Male Mice Suffer More Severe Brain Injury After HI

The overexpression of the *Aif* gene was confirmed at the mRNA level using RNA seq and RT-qPCR analysis. FPKM data from RNA-seq analysis showed that the transcription of *Aif* in AIF hTg mice was 3.2 times higher than in WT mice under physiological conditions (42.10 ± 0.56 FPKM in AIF hTg mice vs. 12.86 ± 0.19 FPKM in WT mice, *p* < 0.0001) (Fig. 1b). The relative abundance of *Aif* transcript variant 1 (*Aif1*) and *Aif* transcript variant 2 (*Aif2*) in WT and AIF hTg mice was determined at 24 h post-HI (Fig. 1c). The total transcription of *Aif* (including both *Aif1* and *Aif2*) in AIF hTg mice was 4.6 times higher than in WT mice (4.63 ± 0.10 in AIF hTg mice vs. 1.00 ± 0.02 in WT mice, *p* < 0.0001). *Aif1* mRNA expression in AIF hTg mice was 10.2 times higher than in WT mice (10.18 ± 0.29 in AIF hTg mice vs. 1.00 ± 0.04 in WT mice, *p* < 0.0001). However, there were no significant differences in *Aif2* mRNA expression between AIF hTg mice and WT mice (1.00 ± 0.04 in AIF hTg mice vs. 1.01 ± 0.05 in WT mice, *p* = 0.8333). No significant differences were found between males and females in WT and AIF hTg mice (Fig. 1c).

**Fig. 2:**
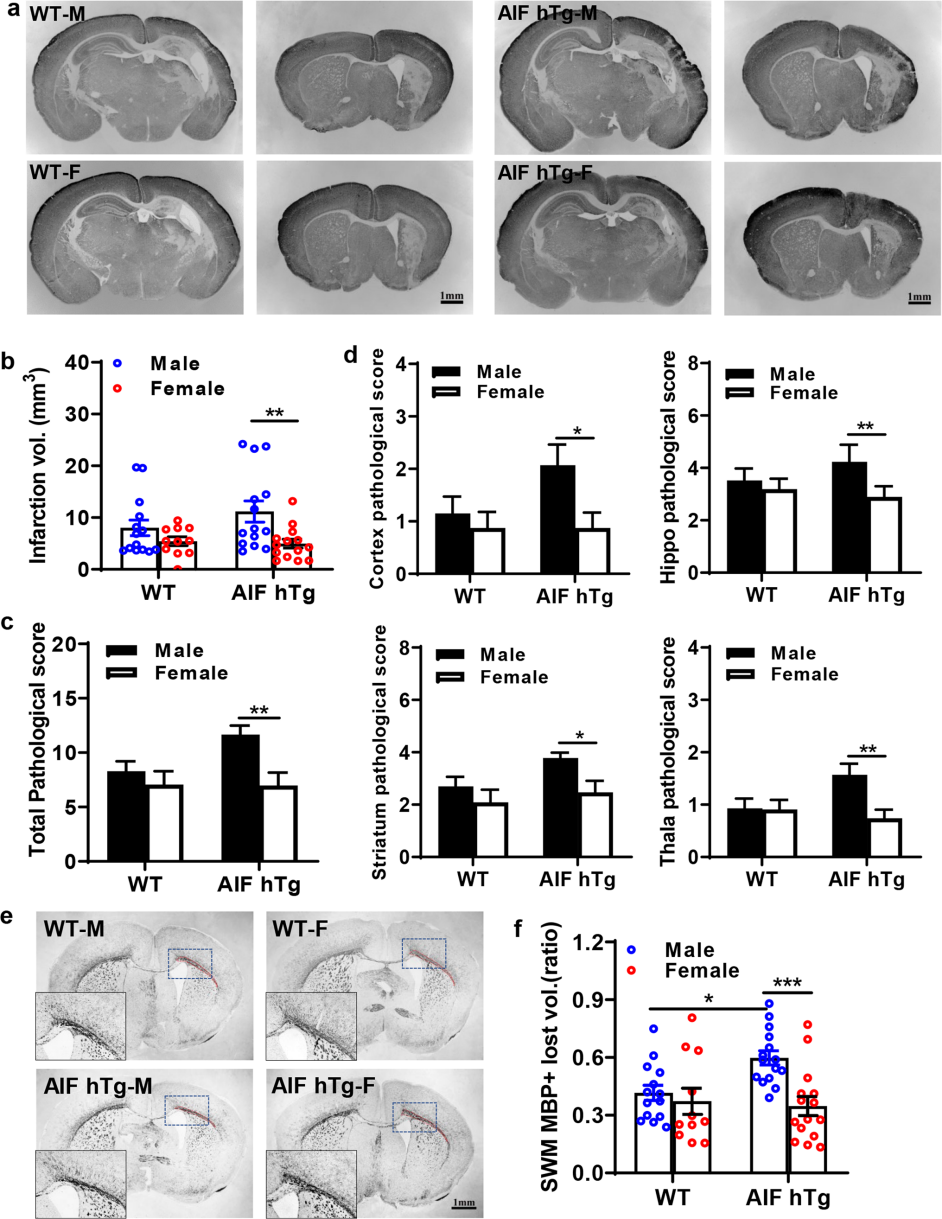
AIF overexpression leads to more severe brain injury in male mice after HI compared to female mice. **a** Representative MAP2 staining of coronal brain Sects. 72 h after HI in both sexes of WT and AIF hTg mice. The upper and lower left panels are the WT male and WT female groups at the dorsal and striatum level, respectively, and the upper and lower right panels are the AIF hTg male and AIF hTg female groups at the dorsal and striatum level, respectively. **b** The infarction volume was measured, and the total pathological score **(c)** and scores of different brain regions **d**, including the cortex (Cx), hippocampus (Hip), striatum (Str), and thalamus (Tha), were evaluated in both sexes of WT mice (*n* = 25, 14 males and 11 females) and AIF hTg mice (*n* = 28, 14 males and 14 females). **e**, **f** Representative MBP staining of coronal brain sections revealed the SWM area (surrounded by the red dashed line) in the IL hemisphere, and the tissue loss ratio in the SWM was quantified in both sexes of WT mice (*n* = 25, 14 males and 11 females) and AIF hTg mice (*n* = 30, 15 males and 15 females). Data are presented as the mean ± SEM and were analyzed using two-way ANOVA followed by Sidak’s post hoc test. *p < 0.05, **p < 0.01, ***p < 0.001

Immunohistochemistry was used to evaluate HI-induced gray matter injury (MAP2) and white matter injury (MBP) in neonatal mice at 72 h after HI. Different regions of brain coronal sections indicated by MAP2 staining were evaluated, including the cortex, hippocampus, striatum, and thalamus (Fig. 2a). Compared with the WT group, AIF overexpression only increased the extent of HI brain injury in AIF hTg male mice (8.02 ± 1.52 mm^3^ in WT males vs. 11.18 ± 2.03 mm^3^ in AIF hTg males, *p* = 0.2315) and not in AIF hTg female mice (5.43 ± 0.84 mm^3^ in WT females vs. 4.97 ± 0.86 mm^3^ in AIF hTg females, *p* = 0.9715) (Fig. 2b). In the AIF hTg mice, the severity of brain injury after HI was significantly greater in males than in females. The extent of gray matter injury in males was 2.25 times higher than in females (11.18 ± 2.03 mm^3^ vs. 4.97 ± 0.86 mm^3^, respectively, *p* = 0.0066), while no significant difference was found between male and female WT mice (8.02 ± 1.52 mm^3^ vs. 5.43 ± 0.84 mm^3^, respectively, *p* = 0.4096) (Fig. 2b). The total neuropathological score was also significantly higher for AIF hTg male mice compared to female mice (11.66 ± 0.84 vs 6.98 ± 1.18, *p* = 0.0038) (Fig. 2c). The pathological score of different brain regions showed that AIF overexpression in males significantly increased HI-induced brain injury compared with AIF hTg female mice (cortex: 2.07 ± 0.39 vs. 0.88 ± 0.29, *p* = 0.0241; hippocampus.: 4.23 ± 0.18 vs 2.89 ± 0.41, *p* = 0.0034; striatum: 3.79 ± 0.20 vs 2.47 ± 0.44, *p* = 0.0289; and thalamus: 1.57 ± 0.21 vs. 0.74 ± 0.16, *p* = 0.0045) (Fig. 2d). Myelination was visualized in the SWM by MBP staining at 72 h after HI (Fig. 2e). The quantification of MBP staining was consistent with the MAP2 results. Thus, the total lost volume of SWM in the ipsilateral hemisphere was greater in the AIF hTg male mice compared to the AIF hTg female mice at 72 h after HI (0.60 ± 0.04 vs. 0.35 ± 0.05, *p* = 0.0006). AIF overexpression significantly increased the loss of volume in the SWM after HI in AIF hTg male mice compared to WT male mice (0.60 ± 0.04 vs 0.42 ± 0.04, *p* = 0.0154). Still, no significant difference was found between AIF hTg female mice and WT female mice (0.35 ± 0.05 vs 0.37 ± 0.07, *p* = 0.9231) (Fig. 2f).

### AIF hTg Male Mice Exhibit Exacerbated Neuronal Cell Death After HI

**Fig. 3:**
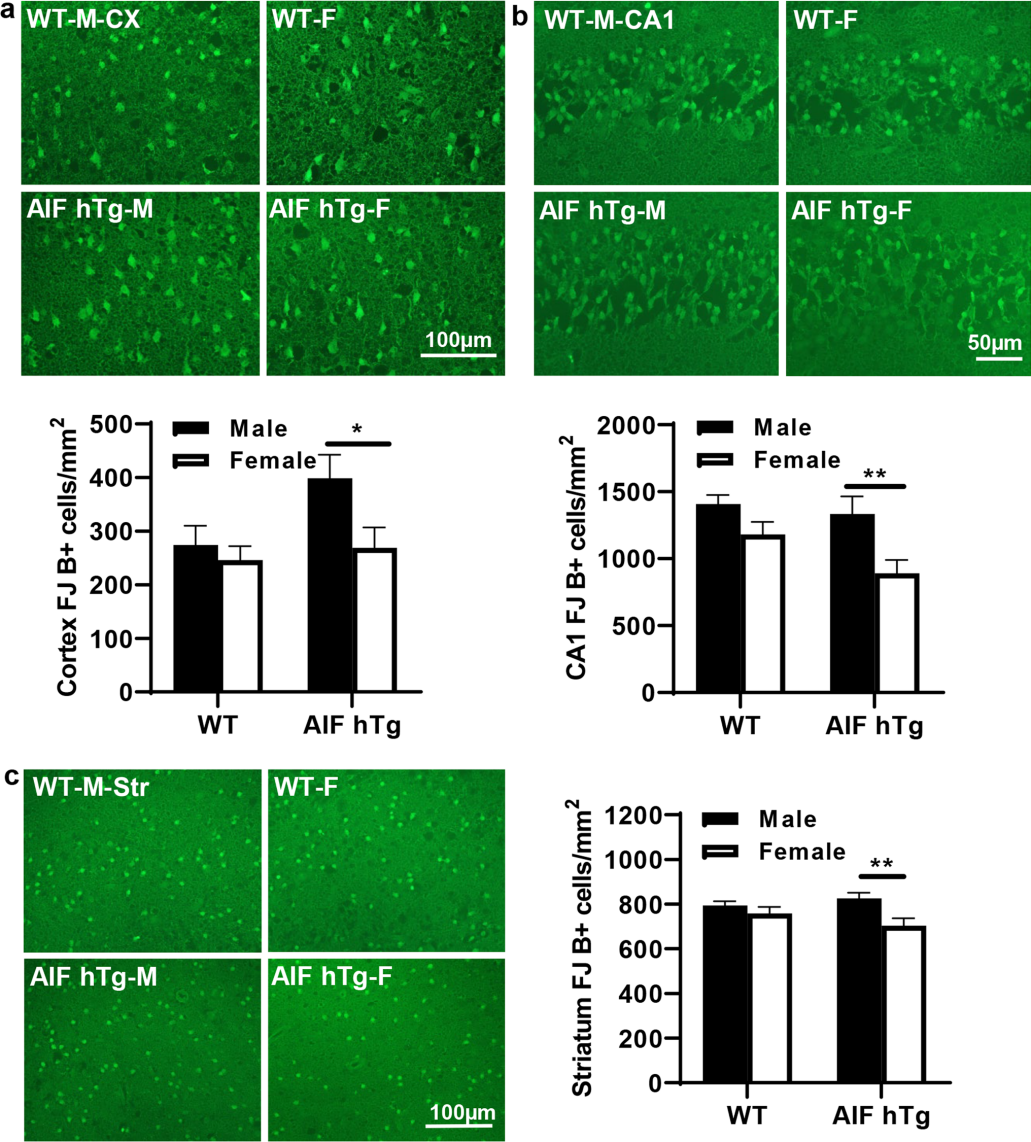
Neuronal cell death in both sexes of WT and AIF hTg mice after HI. Representative images of **a** the cortex, **b** the hippocampal CA1, and **c** the striatum by Fluoro-Jade staining at 24 h post-HI (upper panels), and quantification of Fluoro-Jade-positive cells in both sexes of WT and AIF hTg mice (lower panels) (*n* = 8/group). Cx = cortex, CA1 = cornus ammonis 1, Str = striatum. Data are presented as the mean number of Fluoro-Jade-positive cells ± SEM and were analyzed using two-way ANOVA followed by Sidak’s post hoc test. *p < 0.05, **p < 0.01

Neuronal cell death was detected by Fluoro-Jade staining, a high-affinity fluorescent marker for neuronal degradation, at 24 h after HI. The Fluoro-Jade-positive cells were counted in the cortex, CA1, and striatum. In AIF hTg mice, the numbers of Fluoro-Jade-positive cells in all of these regions were significantly higher in males than in females (Fig. 3a–c). The number of Fluoro-Jade-positive cells in the cortex of the males was 1.48 times greater than in females (398.58 ± 43.77 cells/mm^2^ vs. 268.70 ± 38.38 cells/mm^2^, *p* = 0.0370) (Fig. 3a), 1.50 times greater in the CA1 (1334.09 ± 130.83 cells/mm^2^ vs. 889.97 ± 99.76 cells/mm^2^, *p* = 0.0081) (Fig. 3b), and 1.17 times greater in the striatum (825.29 ± 26.10 cells/mm^2^ vs. 703.98 ± 32.86 cells/mm^2^, *p* = 0.0081) (Fig. 3c). In contrast, no significant differences were found between males and females in the WT group (cortex: 274.06 ± 35.83 cells/mm^2^ vs. 246.18 ± 25.57 cells/mm^2^, *p* = 0.8259; CA1: 1407.81 ± 66.96 cells/mm^2^ vs. 1180.57 ± 93.47 cells/mm^2^, *p* = 0.2266; striatum: 794.29 ± 18.29 cells/mm^2^ vs. 757.72 ± 30.10 cells/mm^2^, *p* = 0.5817).

### AIF hTg Males Exhibit Elevated Apoptotic Cell Death in the CA1 Region

**Fig. 4:**
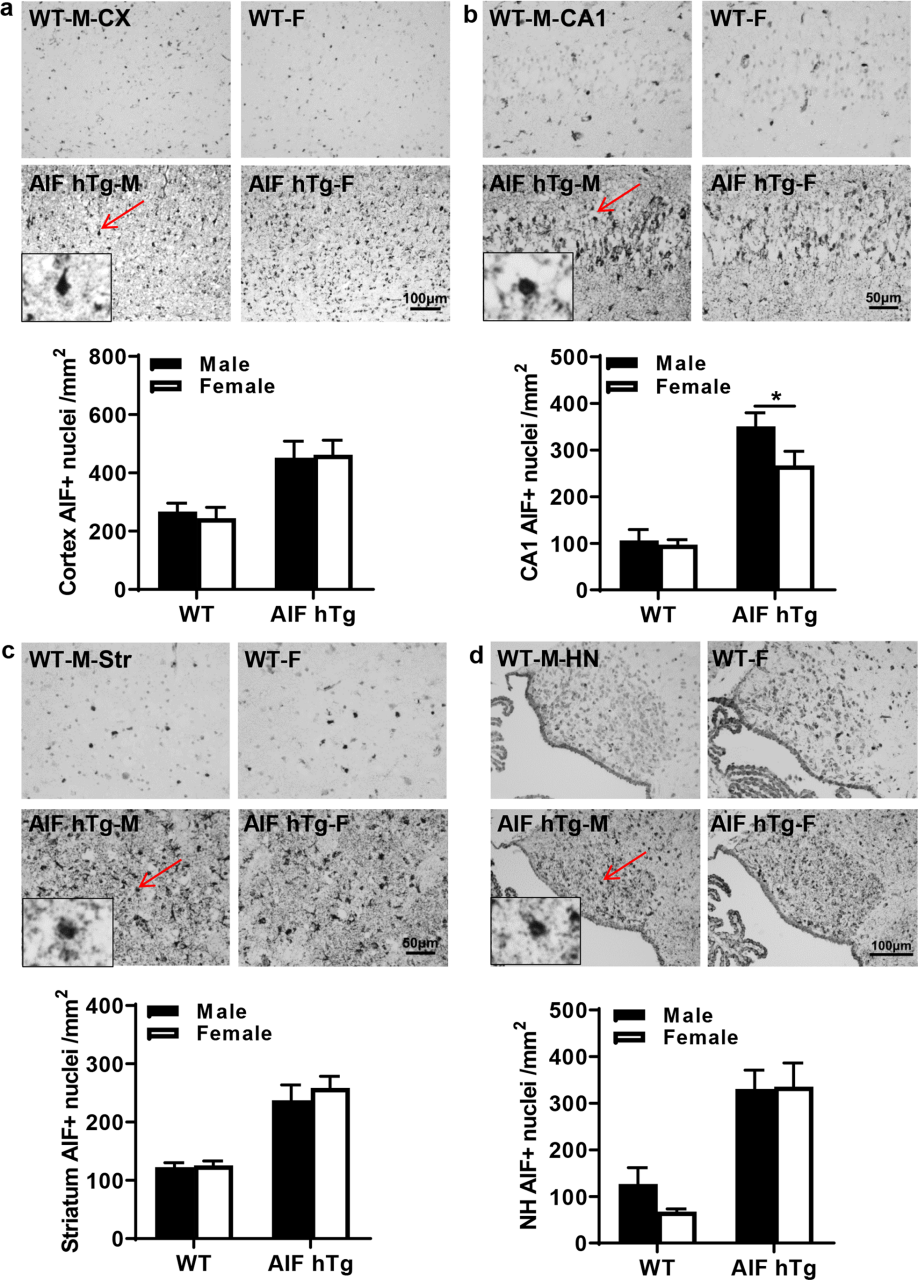
AIF nuclear translocation in different brain regions in both sexes of WT and AIF hTg mice after HI. Representative images of **a** the cortex, **b** the hippocampal CA1, **c** the striatum, and **d** the habenular nuclei by AIF staining at 24 h post-HI (upper panels) and quantification of AIF-positive nuclei in both sexes of WT and AIF hTg mice (lower panels) (*n* = 8/group). Cx, cortex; CA1, cornus ammonis 1; Str, striatum; HN, habenular nuclei. Data are presented as the mean number of AIF-positive nuclei ± SEM and were analyzed using two-way ANOVA followed by Sidak’s post hoc test. *p < 0.05

We evaluated caspase-dependent and independent pathways comparing males and females at 24 h after HI by immunohistochemical detection of AIF and caspase-3. AIF nuclear translocation was significantly increased in several brain regions of both male and female AIF hTg mice at 24 h post-HI (Fig. 4a–d), which is consistent with our previous study. The number of AIF-positive nuclei in AIF hTg males was 1.31 times greater than in females in the CA1 region (350.99 ± 29.00 cells/mm^2^ vs. 266.98 ± 30.45 cells/mm^2^, *p* = 0.0440) (Fig. 4b), but no significant differences were detected in other regions between the male and female AIF hTg mice (Fig. 4a, c, d).

**Fig. 5:**
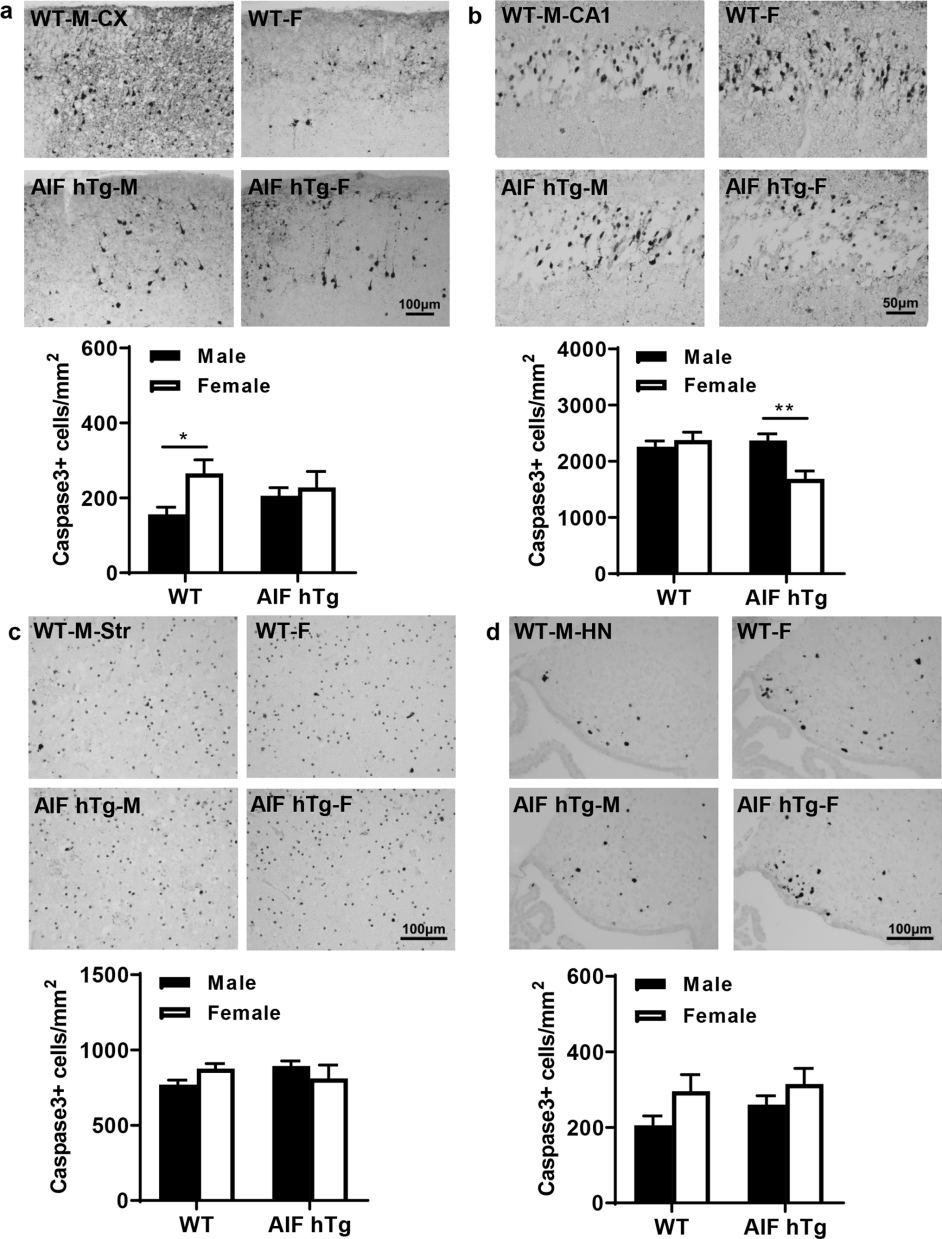
Caspase-3-positive cells in different brain regions in both sexes of WT and AIF hTg mice after HI. Representative images of **a** the cortex, **b** the hippocampal CA1, **c** the striatum, and **d** the habenular nuclei by active caspase-3 staining at 24 h post-HI (upper panels), and quantification of active caspase-3-positive cells in both sexes of WT and AIF hTg mice (lower panels) (*n* = 8/group). Cx, cortex CA1, cornus ammonis 1; Str, striatum; HN, habenular nuclei. Data are presented as the mean number of active caspase-3-positive cells ± SEM and were analyzed using two-way ANOVA followed by Sidak’s post hoc test. *p < 0.05, **p < 0.01

The numbers of active caspase-3-positive cells were counted by immunostaining in different brain regions at 24 h after HI, which is when caspase-3 activation reaches its peak (Fig. 5a–d). In all observed brain regions of WT mice, the number of caspase-3-positive cells was higher in females than in males, especially in the cortex. The number of caspase-3-positive cells in the cortex of females was 1.70 times greater than in males (265.50 ± 36.37 cells/mm^2^ vs. 156.12 ± 19.11 cells/mm^2^, *p* = 0.0429) (Fig. 5a). However, in AIF hTg mice the number of caspase-3-positive cells in the CA1 of the females was significantly lower than in males (1690.48 ± 138.49 cells/mm^2^ vs. 2369.05 ± 121.75 cells/mm^2^, *p* = 0.0014) (Fig. 5b), and the number in the striatum of females was slightly lower than in males (811.51 ± 88.17 cells/mm^2^ vs. 894.34 ± 32.37 cells/mm^2^, *p* = 0.8412) (Fig. 5c). No significant difference was found between AIF hTg male mice and female mice in the habenular nuclei area (260.37 ± 23.24 cells/mm^2^ vs. 314.97 ± 41.28 cells/mm^2^, *p* = 0.5006) (Fig. 5d). Thus, the reduced HI-induced brain injury in AIF hTg female mice might be related to attenuated apoptotic cell death in the CA1 region.

### AIF hTg Male Mice Have Greater Oxidative Stress Responses After HI

**Fig. 6:**
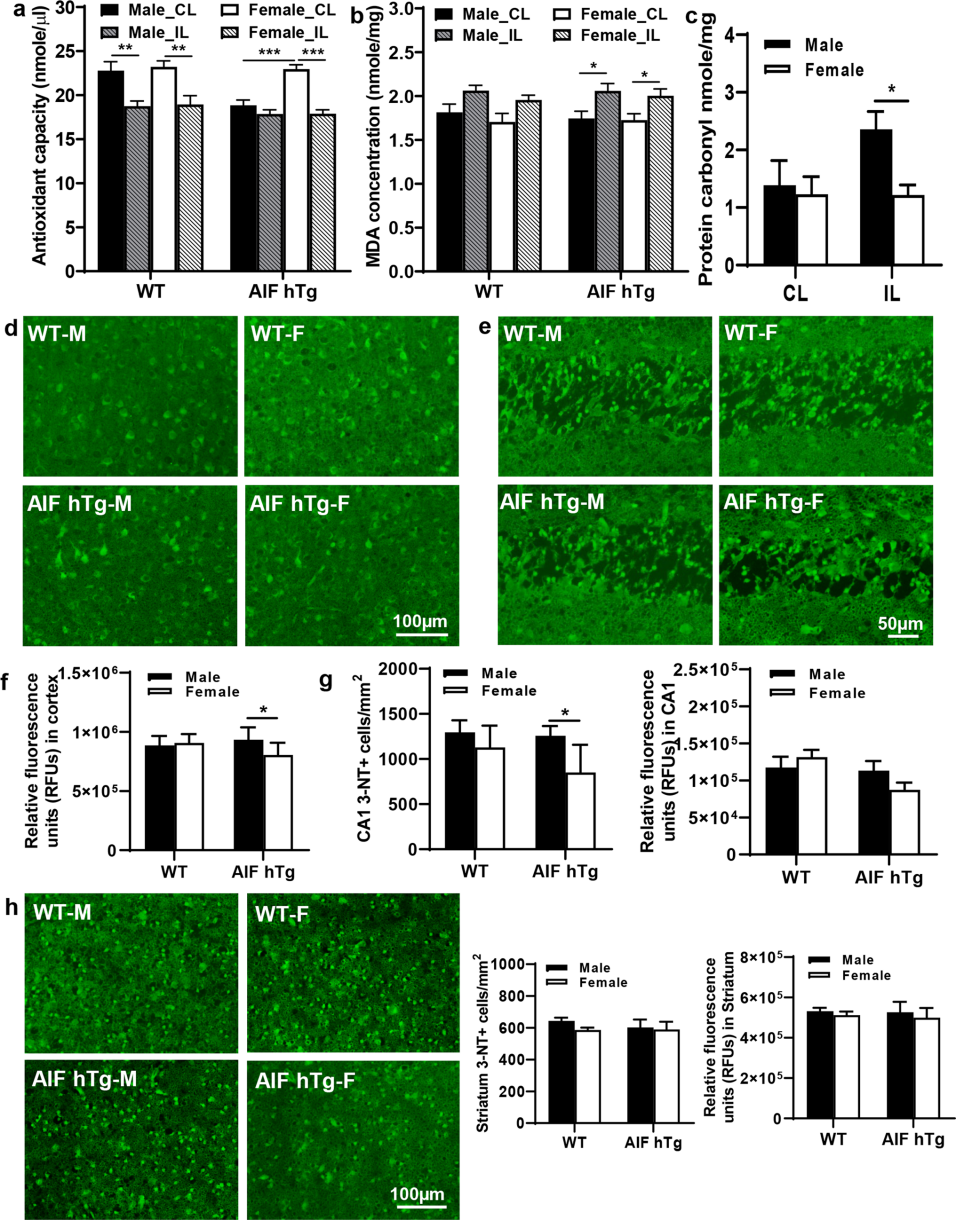
Oxidative stress responses were different between male and female AIF hTg mice after HI. The concentrations of total antioxidants (**a**) and MDA (**b**) in homogenates of cortical brain tissues were measured in males and females of both WT and AIF hTg mice using an ELISA kit (*n* = 6/group). The protein carbonylation (**c**) was determined further in both the CL hemisphere and IL hemisphere in both male and female AIF hTg mice using an ELISA kit (*n* = 6/group). Immunofluorescence staining was performed to determine the number of 3-NT-positive cells and RFUs of the 3-NT-positive area. **d**, **f** Representative 3-NT staining in the cortex in both sexes of WT and AIF hTg mice at 24 h post-HI and quantification of the RFUs of the 3-NT-positive area (*n* = 8/group). Representative 3-NT staining in CA1 (**e**, **g**) and the striatum (**h**) in both sexes of WT and AIF hTg mice at 24 h post-HI and quantification of the 3-NT-positive cells and RFUs of the 3-NT-positive area (*n* = 8/group). Data are presented as the mean ± SEM and were analyzed using two-way ANOVA followed by Sidak’s post hoc test. *p < 0.05, **p < 0.01, ***p < 0.001

Different indicators of oxidative stress were analyzed in brains at 24 h after HI. Antioxidants can scavenge free radicals generated by oxidants and prevent cellular oxidative stress through both enzymatic and non-enzymatic mechanisms [32]. The total antioxidant capacity of the neonatal brain after HI, including protein antioxidants and small molecule antioxidants, was determined (Fig. 6a). Compared to the contralateral (CL) hemisphere, the concentration of total antioxidants in the ipsilateral (IL) hemisphere was significantly decreased in male and female WT mice at 24 h after HI (for males, 22.78 ± 1.02 nmol/μl in the CL hemisphere vs. 18.76 ± 0.58 nmol/μl in the IL hemisphere, *p* = 0.0063; for females, 23.20 ± 0.69 nmol/μl in the CL hemisphere vs. 18.93 ± 1.01 nmol/μl in the IL hemisphere, *p* = 0.0039). A significant decrease was also found in female AIF hTg mice (22.95 ± 0.50 nmol/μl in the CL hemisphere vs. 17.89 ± 0.44 nmol/μl in the IL hemisphere, *p* < 0.0001), but only a slight decrease was seen in male AIF hTg mice (18.85 ± 0.60 nmol/μl in the CL hemisphere vs. 17.86 ± 0.48 nmol/μl in the IL hemisphere, *p* = 0.9987). Unexpectedly, in the CL hemisphere the concentration of antioxidants in male AIF hTg mice was significantly lower than in females (18.85 ± 0.60 nmol/μl vs. 22.95 ± 0.50 nmol/μl, *p* < 0.0001).

MDA is a lipid peroxidation marker and can be used to measure oxidative stress [33]. The HI insult increased the amount of MDA in all groups at 24 h after HI, especially in AIF hTg mice (for WT males, 1.81 ± 0.09 nmol/mg in the CL hemisphere vs. 2.06 ± 0.06 nmol/mg in the IL hemisphere, *p* = 0.0713; for WT females, 1.71 ± 0.10 nmol/mg in the CL hemisphere vs. 1.96 ± 0.05 nmol/mg in the IL hemisphere, *p* = 0.0685; for AIF hTg males, 1.74 ± 0.08 nmol/mg in the CL hemisphere vs. 2.06 ± 0.08 nmol/mg in the IL hemisphere, *p* = 0.0300; for AIF hTg females, 1.73 ± 0.07 nmol/mg in the CL hemisphere vs. 2.00 ± 0.08 nmol/mg in the IL hemisphere, *p* = 0.0359) (Fig. 6b). However, no significant differences were found between males and females either in WT or AIF hTg mice.

Protein carbonylation, one of the most frequent ROS-induced protein modifications, was measured in male and female AIF hTg mice. The overexpression of AIF did not affect protein carbonylation in males or females in the CL hemisphere (1.39 ± 0.43 nmol/mg vs. 1.23 ± 0.31 nmol/mg, *p* = 0.9274); however, in the IL hemisphere, the level of protein carbonylation in male AIF hTg mice was significantly higher than in female AIF hTg mice at 24 h after HI (2.36 ± 0.31 nmol/mg vs 1.22 ± 0.17 nmol/mg, *p* = 0.0382) (Fig. 6c).

3-nitrotyrosine (3-NT), the main product of tyrosine oxidation, is a valid biomarker of oxidative damage mediated by peroxynitrite. The protein nitration level was determined in the cortex, hippocampus (CA1), and striatum areas at 24 h after HI using 3-NT immunofluorescence staining and was quantified by 3-NT-positive cell numbers and by RFUs. The RFUs of the cortex in male AIF hTg mice were 16.1% higher than in females (9.34 × 10^5^ ± 0.37 × 10^5^ vs. 8.05 × 10^5^ ± 0.39 × 10^5^, *p* = 0.0214) (Fig. 6d, f), but no significant difference was found between male and female WT mice (8.86 × 10^5^ ± 0.28 × 10^5^ vs. 9.06 × 10^5^ ± 0.27 × 10^5^, *p* = 0.8857). The 3-NT-positive cells in the cortex were not counted due to the non-specific cell morphology. In the CA1 of the hippocampus, the number of 3-NT-positive cells in male AIF hTg mice was 1.48 times higher than in females (1256.48 ± 108.90 cells/mm^2^ vs. 850.60 ± 116.28 cells/mm^2^, *p* = 0.0361); however, no significant differences were found between male and female WT mice (1295.44 ± 132.75 cells/mm^2^ vs. 1127.82 ± 85.48 cells/mm^2^, *p* = 0.4988) (Fig. 6e, 6g). The RFUs of the CA1 showed no significant differences between males and females for both WT and AIF hTg mice (*p* = 0.6587 for WT mice, *p* = 0.2539 for AIF hTg mice). In the striatum, neither the number of 3-NT-positive cells nor the RFUs were different between males and females for both WT and AIF hTg mice (for 3-NT-positive cell number, *p* = 0.4296 for WT mice and *p* = 0.9614 for AIF hTg mice; for RFUs, *p* = 0.9103 for WT mice and *p* = 0.8523 for AIF hTg mice) (Fig. 6h). These results suggested that AIF overexpression caused a much stronger oxidative stress response in neonatal male mice after HI insult, especially in the CA1 region.

Mitochondria are an essential source of ROS in most mammalian cells [34], and in neurons, ROS have been shown to regulate mitochondrial dynamics, which involves mitochondrial fission and fusion processes [35]. Thus, changes in fission and fusion-related genes were determined at the mRNA and protein levels (Fig. S2a-2c). After HI, AIF overexpression had no effect on mitochondrial dynamic changes between males and females, except that HI increased mitochondrial fission and decreased mitochondrial fusion. Nevertheless, under physiological conditions, AIF overexpression significantly increased the expression of the fission protein phospho-DRP1 in females compared to males, which could inhibit mitochondrial fission (Fig. S2b, S2c).

### Female AIF hTg Mice Have More Robust Neuronal Proliferation in the Dentate Gyrus Region Under Physiological Conditions

**Fig. 7:**
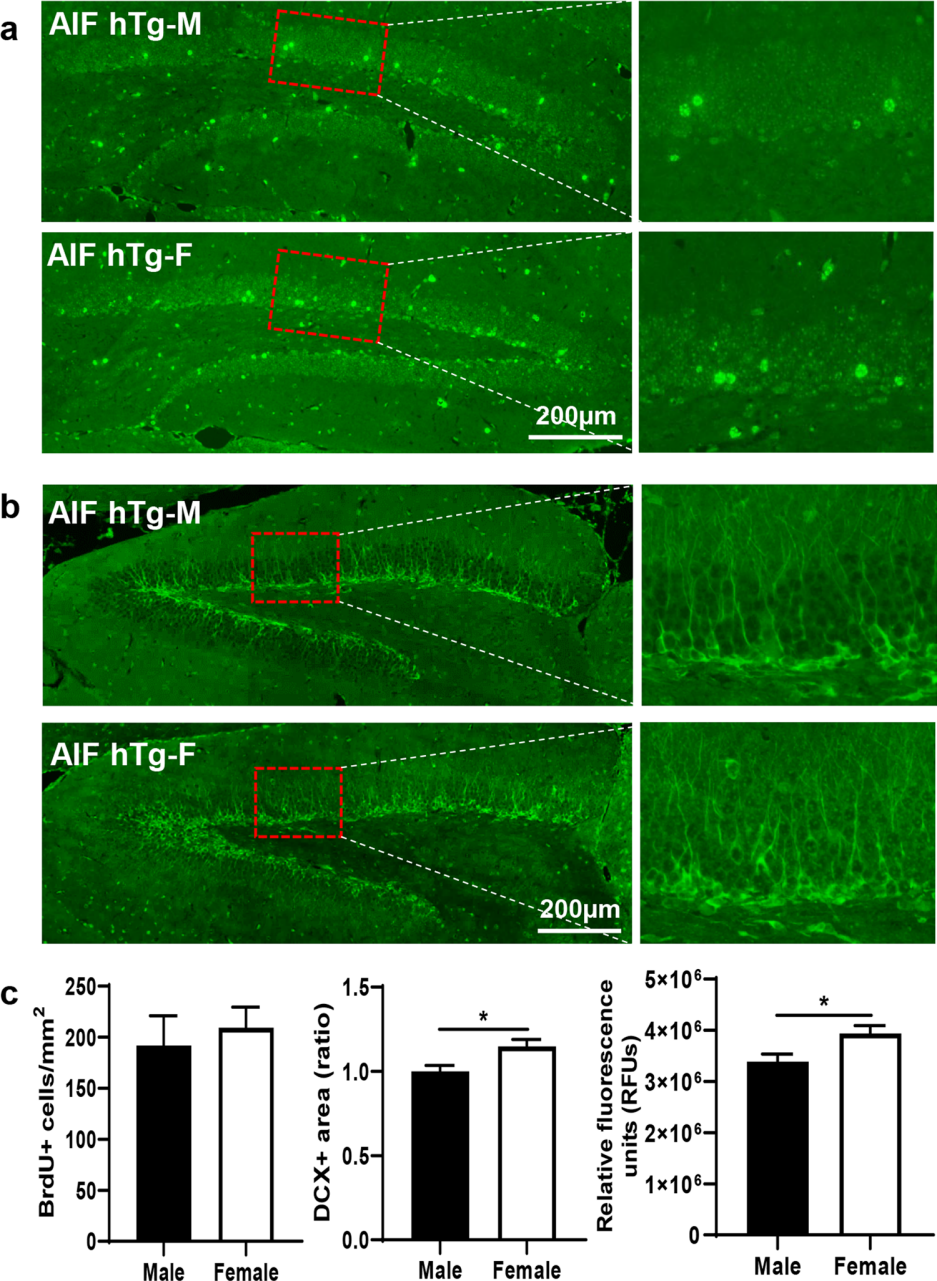
Changes in neuronal proliferation between male and female AIF hTg mice under physiological conditions. Representative panoramic images of BrdU (**a**) and DCX (**b**) staining showing the dentate gyrus in male and female AIF hTg mice at P30. The number of BrdU-positive cells was quantified in the granular layer of the dentate gyrus, and the DCX-positive area and the RFUs of this area were quantified (*n* = 6/group). Data are presented as the mean ± SEM and were analyzed using Student’s *t*-test. *p < 0.05

In this study, we determined the effects of AIF overexpression on neuronal proliferation both in male and female AIF hTg mice by quantifying the numbers of BrdU-positive cells and DCX-positive cells in the granule cell layer of the dentate gyrus by immunofluorescence staining. At P30, no significant differences in BrdU-positive cells were found between male and female AIF hTg mice (191.70 ± 29.21 cells/mm^2^ vs. 209.40 ± 19.99 cells/mm^2^, *p* = 0.6286) (Fig. 7a). However, the DCX-positive area of the granular layer of the dentate gyrus in females was 1.15 times larger than in males (ratio: 1.15 ± 0.04 vs. 1.00 ± 0.04, *p* = 0.0214), and the RFUs of the DCX-positive area in females were also significantly greater than in males (3.94 × 10^6^ ± 1.50 × 10^5^ vs. 3.39 × 10^6^ ± 1.47 × 10^5^, *p* = 0.0255) (Fig. 7b). The BrdU/DCX double-positive cells were further analyzed. However, no significant difference was found between AIF hTg males and females (Fig. S3). Combined with our previous results, this indicated that AIF overexpression could promote neuronal proliferation in a long-term manner and that this effect is more pronounced in females.

### The Effect of AIF Overexpression on Carbohydrate Metabolism Under Physiological Conditions

**Fig. 8:**
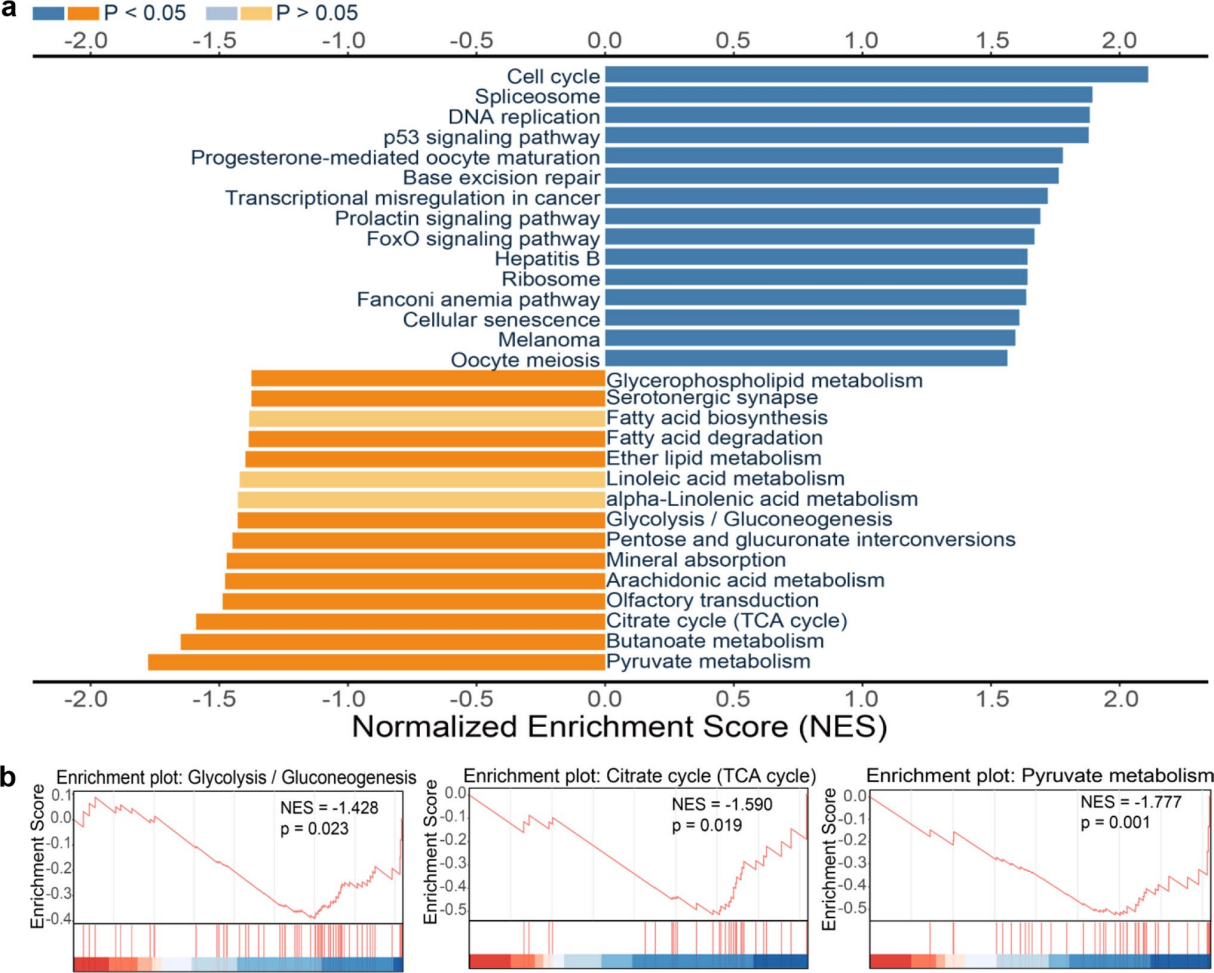
GSEA in the cortex of AIF hTg mice under physiological conditions. **a** Based on the *p*-value, the top 15 positively and negatively enriched KEGG pathways were selected to plot against the normalized enrichment score. Dark blue and deep orange represent *p* < 0.05, and light blue and light orange represent pathways with *p* > 0.05. **b** Energy metabolism-related pathways—including glycolysis (gluconeogenesis), the citrate cycle (TCA cycle), and pyruvate metabolism—were enriched according to GSEA, and the normalized enrichment scores and *p*-values are shown in the enrichment plot

To further investigate the effects of AIF overexpression, we compared the mRNA transcriptome between males and females in both WT groups and AIF hTg groups under physiological conditions. The transcriptomes in the cortex of six P9 male and female mouse brains were determined by RNA sequencing. GSEA was performed to characterize the specific pathways affected by the transcriptome changes between males and females. We found 38 positively enriched pathways and 12 negatively enriched pathways with a *p*-value of < 0.05 in AIF hTg female mice compared to AIF hTg male mice. In the WT group, 26 positively and 5 negatively enriched pathways were found with a *p*-value of < 0.05 in female mice compared to males. The top 15 positively enriched and negatively enriched pathways were plotted against the normalized enrichment score (Fig. 8a, Fig. S4a).

Carbohydrate metabolism is a fundamental biochemical process that ensures a constant supply of energy to living cells [36], and gene expression associated with the main processes of carbohydrate metabolism, including glycolysis, the citrate cycle, and pyruvate metabolism, was reduced in female AIF hTg mice compared with males (Fig. 8b). However, the main energy metabolism pathways, including oxidative phosphorylation, the citrate cycle, and carbon metabolism showed a positive trend in WT female mice compared with males (Fig. S4b). AIF overexpression may have different effects on energy metabolism in males and females under physiological conditions. All of these results indicated that female AIF hTg mice had reduced energy supply compared to males under physiological conditions, and this may be related to the reduced brain injury seen in female AIF hTg mice after HI.

## Discussion

Our previous study in mice and clinical studies have shown sex differences in the severity, mechanisms, outcomes, and medication responses in neonatal brain injury after HI [26, 37–39]. The male sex is considered to be a risk factor for unfavorable outcomes of HIE [27], but the mechanisms behind this sex-related difference are not well understood. A previous study indicated that different apoptotic mechanisms are activated in male and female brains after HI [37]. In neonatal mice after HI insult, the AIF-mediated caspase3-independent cell death pathway is more prominent in males, whereas caspase3-dependent cell death is more pronounced in females. The results of infarction and SWM volume showed that male AIF hTg mice had more severe brain injuries than females after HI, and this might be related to the greater neuronal cell death in males after AIF overexpression. The severity of HI-induced brain injury in AIF hTg male mice was much higher than in the WT male mice; however, the HI-induced infarction volume of AIF hTg female mice was slightly smaller than the WT female mice. These results indicated that the upregulation of AIF has a prominent effect on males after HI but only slightly impacts females.

Different regions of the developing brain have distinct vulnerabilities to hypoxic–ischemic damage [40], and this might be related to the severity of excitotoxicity, oxidative stress, inflammatory response, and cerebrovascular effects [41]. Also, different cell populations and cell types might undergo different cell death pathways in different brain regions [30, 42]. The hippocampus is a brain region critical for memory consolidation and spatial navigation, and the CA1 neurons of the hippocampus are known to be especially vulnerable to ischemic insults [43]. Sex differences have been reported in terms of hippocampal plasticity and cognition in many disorders that target the integrity of the hippocampus [44, 45]. In the present study, AIF upregulation significantly increased the caspase-3-independent cell death pathway in all brain regions of both AIF hTg male and female mice compared with WT mice. However, significant differences in AIF nuclear translocation and caspase-3 activation between AIF hTg males and females were only detected in the CA1 region. These results suggest that AIF overexpression makes the neuronal apoptotic cell death caused by HI injury in the hippocampus of males more pronounced than in females.

Enhanced oxidative stress was detected in dying neurons in Hq mice leading Klein et al. to propose that AIF might act as a free radical scavenger to inhibit apoptosis [20], and other studies have further supported this hypothesis [19, 46]. However, the potential free radical scavenging function of AIF is still controversial because of conflicting results in different experimental models [19, 47, 48]. In our AIF upregulation mouse model of neonatal HI injury, more severe brain injury was found in males. However, in this study the upregulation of AIF did not reinforce free radical scavenging but instead significantly decreased the antioxidant capacity in males, leading to enhanced oxidative stress and more severe brain injury under pathological conditions.

Compared to the adult brain, the weak antioxidant defense mechanisms of the neonatal brain sensitize the tissue to oxidative stress. Hence, HI can increase the levels of free radicals and disrupt redox homeostasis in the brain, thus resulting in neuronal cell death [49]. MDA levels notably increased after HI in both WT and AIF hTg males and females. However, no significant differences were seen between AIF hTg males and females, which indicated that AIF overexpression did not affect lipid peroxidation after HI brain injury in neonates. Higher levels of protein carbonylation and 3-NT modification in different brain regions, especially CA1, were seen in AIF hTg mice, and these results suggest that AIF upregulation led to a weak antioxidative response in males and thus to more severe oxidative stress and increased protein carbonylation and nitration after HI. Sex-specific effects of oxidative stress have also been shown to exist in newborn humans [50]. Compared to girls during the neonatal period, all markers of sex-dependent oxidative stress are higher in boys and all markers associated with antioxidant defense are lower [51]. Although we did not find the markers used in this study to show a significant difference between males and females in the WT group, it seems that AIF upregulation makes these sex differences in oxidative stress more pronounced. Neuronal mitochondria are considered to be a sensor of oxidative stress during neuronal development [49], and oxidative stress can induce mitochondrial dysfunction leading to further oxidative stress responses [52]. We found that the mitochondrial dynamic balance was disturbed after HI in this study, which is consistent with our previous results, but AIF upregulation does not appear to change the mitochondrial dynamics between males and females.

AIF is critical for mitochondrial bioenergetics. In Hq mice, AIF deficiency is proposed to disrupt oxidative phosphorylation [20], and conditional knockout of AIF has been shown to cause defects in oxidative phosphorylation complex I in skeletal muscles [53]. Carbohydrate metabolism—including glycolysis, gluconeogenesis, the citrate cycle, pyruvate metabolism, and the pentose phosphate pathway—is the fundamental biochemical process responsible for the metabolic formation, breakdown, and interconversion of carbohydrates in living organisms [36]. Under physiological conditions, our GSEA results indicated that the pathways related to carbohydrate metabolism, including glycolysis, the citrate cycle, and pyruvate metabolism, were down-regulated in female AIF hTg mice. These results suggest that AIF upregulation can significantly promote bioenergetic pathways in males, and these enhanced metabolic pathways might cause the males to suffer from greater oxidative stress after HI.

A previous study reported that AIF deficiency results in cell type-specific neurogenesis defects in Hq mice [54], and AIF has been shown to be indispensable for cell type-specific neurogenesis in the prospective midbrain and cerebellum at very early stages of brain development [20]. Our previous study showed that excessive AIF might increase neuronal proliferation in the long term [30]. In the present study, sex differences in neurogenesis were further investigated in AIF hTg mice. The number of DCX-positive cells in the granular layer of the dentate gyrus of females at 1 year of age was significantly higher than in males, but no difference in BrdU-positive cells was seen in the same brain area at P30 between males and females. These results suggested that AIF upregulation had a more pronounced long-term effect on cell proliferation or cell loss in female AIF hTg mice but did not impact the early stages of brain development.

There are some limitations to the current study that should be noted. First, due to the low frequency of the homozygous genotype, which limited the number of mice obtained, samples from early time points, such as 6 h and 12 h post-HI, could not be obtained. Second, even though carbohydrate metabolism-related pathways were down-regulated in female AIF hTg mice under physiological conditions, we did not obtain transcriptome data after HI. We found that AIF overexpression in males aggravated brain injury because of higher oxidative stress, but this finding would be more convincing if transcriptomic and proteomic analyses were performed post-HI.

## Conclusion

Our study demonstrates that AIF upregulation significantly aggravates HI-induced brain injury in neonatal males compared to female mice, and this is associated with increased neuronal cell death, activation of apoptotic cell death, reduced antioxidant capacity, and enhanced oxidative stress. In the long term, AIF upregulation had a pronounced effect on cell proliferation in females but did not impact the early stages of brain development. Altogether, this study indicates that AIF is involved in the antioxidative and oxidative stress responses in neonatal hypoxic–ischemic brain injury and has a long-term neurogenic effect, with pronounced sex differences after HI brain injury in neonatal mice.

## Supplementary Information

Below is the link to the electronic supplementary material.

Supplementary file1 (PDF 841 KB)

## Data Availability

All data generated or analyzed during this study are included in this published article and its supplementary information files.

## Abbreviations

HI: Hypoxia–ischemia
AIF: Apoptosis-inducing factor
HIE: Hypoxic–ischemic encephalopathy
ROS: Reactive oxygen species
MAP2: Microtubule-associated protein 2
MBP: Myelin basic protein
RFUs: Relative fluorescence units
CA1: Cornus ammonis 1
DRP1: Dynamin-related protein 1
FIS1: Mitochondrial fission 1 protein
OPA1: Optic atrophy protein 1
VDAC1: Voltage-dependent anion-selective channel 1
MDA: Malondialdehyde
GSEA: Gene set enrichment analysis
SWM: Subcortical white matter
CL: Contralateral hemisphere
IL: SsssIpsilateral hemisphere
