# Supplementary material for: AIF Overexpression Aggravates Oxidative Stress in Neonatal Male Mice After Hypoxia–Ischemia Injury
Source: Mol Neurobiol. 2022 Aug 17;59(11):6613–31. doi: 10.1007/s12035-022-02987-0 (PMC9525408; doi:10.1007/s12035-022-02987-0)
Supplement: Supplementary file 1 — Supplementary file1 (PDF 841 KB) [file 12035_2022_2987_MOESM1_ESM.pdf]

# Fig. S1

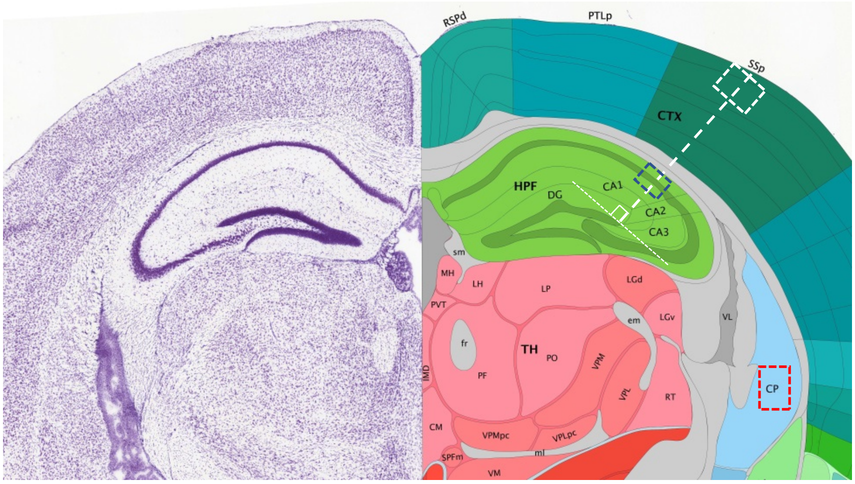


**Fig. S1. Schematic figure of area selections in brain sections**. For the cortex and CA1 selection, the area was first located based on the relative vertical distance to the granular layer of the dentate gyrus (DG). This is indicated with two vertical white dotted lines. The white dashed square indicates the selected area in the cortex. The blue dashed square indicates the selected area in CA1. For the striatum selection, the red dashed square in the middle of the caudate putamen (CP) indicates the selected area in the striatum. Image credit: Allen Institute for Brain Science. [http://atlas.brain-map.org/atlas?atlas=1&plate=100960224]

# Fig. S2

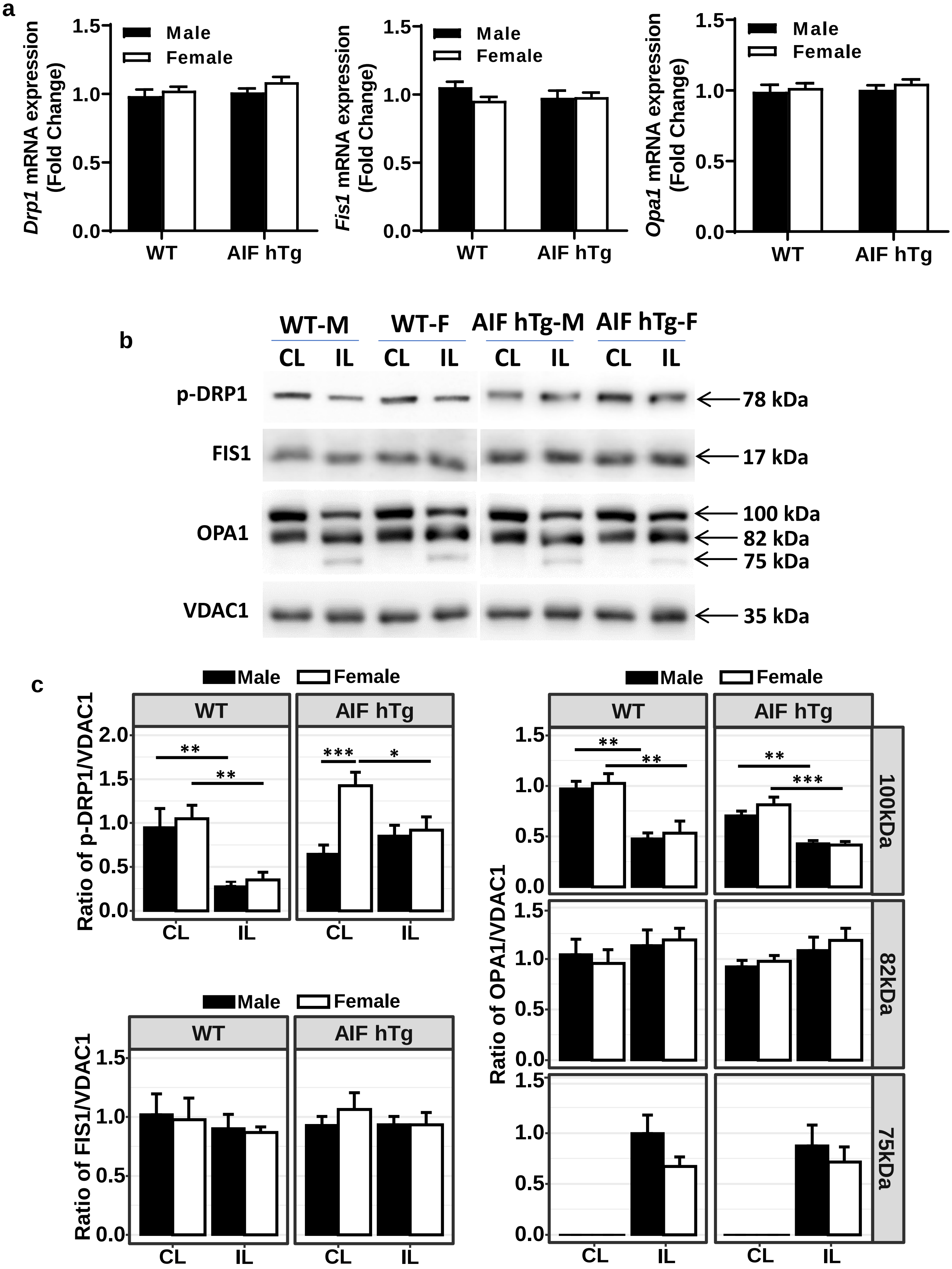

a
Male
Female
Drp1 mRNA expression (Fold Change)
Fis1 mRNA expression (Fold Change)
Opa1 mRNA expression (Fold Change)
0.0
0.5
1.0
1.5
WT
AIF hTg
b
WT-M
WT-F
AIF hTg-M
AIF hTg-F
CL
IL
p-DRP1
FIS1
OPA1
VDAC1
78 kDa
17 kDa
100 kDa
82 kDa
75 kDa
35 kDa
c
Ratio of p-DRP1/VDAC1
Ratio of FIS1/VDAC1
Ratio of OPA1/VDAC1
2.0
100kDa
82kDa
75kDa

**Fig S2. AIF overexpression has no effects on mitochondrial dynamics between males and females.**

**(a)** The mRNA expressions of *Drp1*, *Fis1*, and *Opa1* were determined by RT-qPCR in cortical tissue of WT and AIF hTg mice at 24 h after HI (n = 6/group). **(b)** Representative immunoblotting of p-DRP1, FIS1, and OPA1 in the mitochondrial fraction of cortical tissue from the CL and IL hemispheres of both sexes of WT and AIF hTg mice at 24 h after HI (n = 6/group). **(c)** Quantification of p-DRP1, FIS1, and OPA1 did not show any significant differences between the males and females in the IL hemisphere of both WT and AIF hTg mice at 24 h after HI. However, comparing the CL and IL hemispheres, the expression of p-DRP1 was significantly reduced in the IL hemisphere in both males and females of WT mice ($p = 0.0080$ for males, $p = 0062$ for females). In AIF hTg mice, the reduction in the IL hemisphere was only found in females ($p = 0.0322$), which may be related to the significant increase in the CL hemisphere compared with male AIF hTg mice ($p = 0.0010$). The long form of OPA1 was significantly reduced in both WT and AIF hTg mice at 24 h after HI ($p = 0.0016$ for male WT mice, $p = 0.0017$ for female WT mice, $p = 0.0016$ for male AIF hTg mice, $p < 0.0001$ for female AIF hTg mice); however, the 82 kDa short form of OPA1 was increased ($p = 0.8828$ for male WT mice, $p = 0.4361$ for female WT mice, $p = 0.4424$ for male AIF hTg mice, $p = 0.2730$ for female AIF hTg mice), and the 75 kDa short form of OPA1 was detected at 24 h after HI in the two genotypes of mice. Data are presented as the mean ± SEM and were analyzed using two-way ANOVA followed by Sidak's post hoc test. * $p < 0.05$, ** $p < 0.01$, *** $p < 0.001$.

# Fig. S3

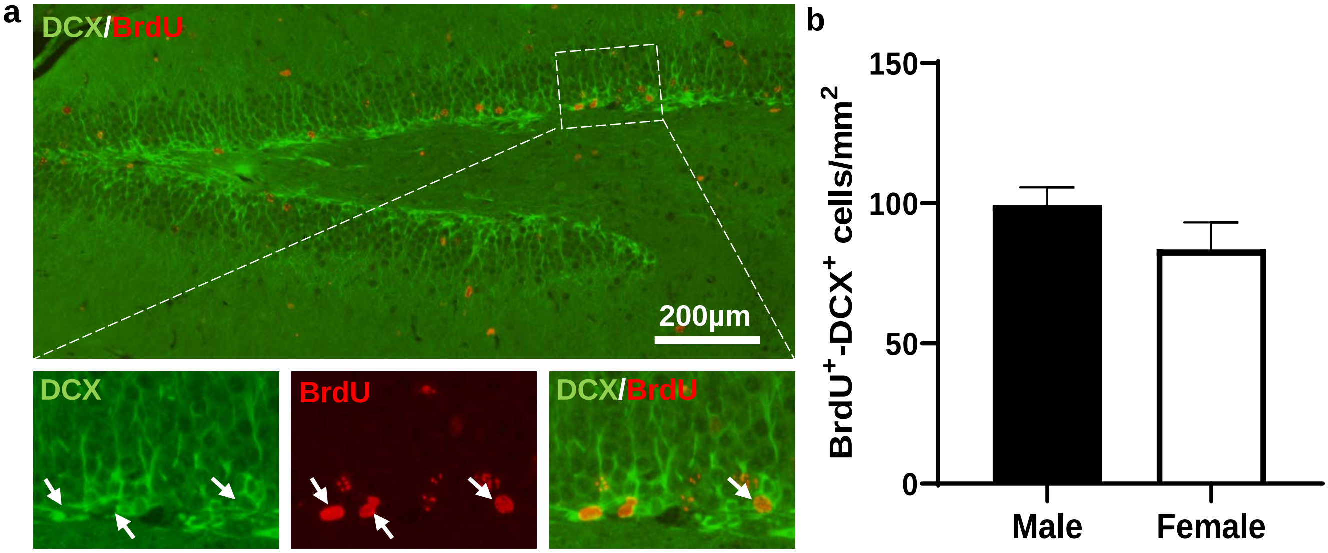


**Fig. S3 BrdU/DCX double-positive cells between male and female AIF hTg mice under physiological conditions.**
**(a)** Representative panoramic images of BrdU-DCX double staining showing the dentate gyrus. **(b)** The number of BrdU-DCX double positive cells were counted, and it was slightly higher in AIF hTg male mice compared to AIF hTg female mice, but without a statistical difference (99.48 ± 6.15 cells/$mm^2$ in AIF hTg male mice, 83.53 ± 9.69 cells/$mm^2$ in AIF hTg female mice, $p = 0.214$). Data are presented as the mean ± SEM and were analyzed using Student's t-test.

# Fig. S4

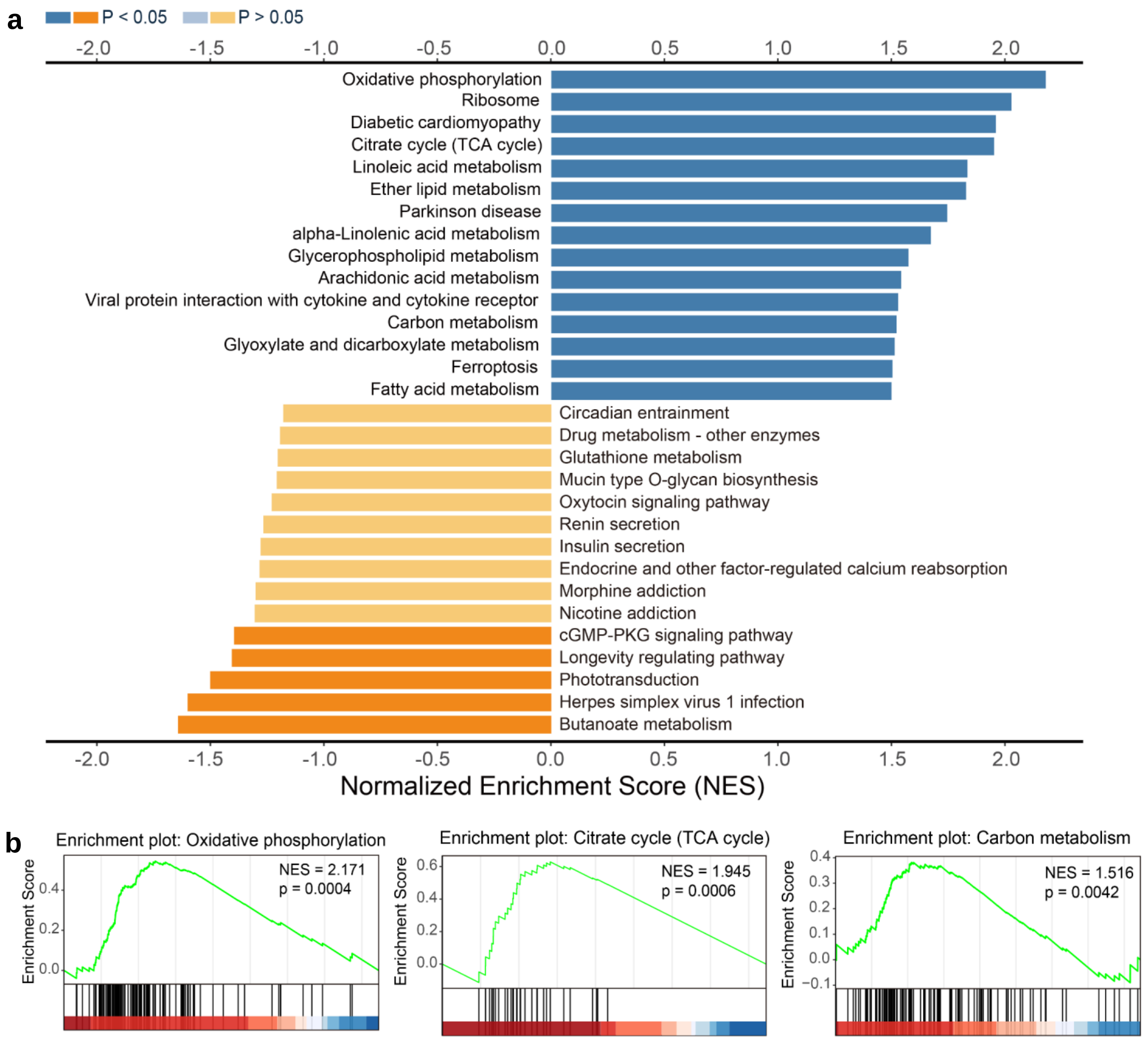


**Fig S4. GSEA in the cortex of WT mice under physiological conditions.**

**(a)** The top 15 positively and negatively enriched KEGG pathways were selected to plot against the normalized enrichment score. Dark blue and deep orange represent $p < 0.05$, and light blue and light orange represent pathways with $p > 0.05$. **(b)** Energy metabolism-related pathways – including oxidative phosphorylation, the citrate cycle (TCA cycle), and carbon metabolism – were enriched according to GSEA, and the normalized enrichment scores and p-values are shown in the enrichment plot.
